# Proof-of-concept study on improved efficacy of rHuEPO administered as a long-term infusion in rats

**DOI:** 10.1007/s43440-020-00150-x

**Published:** 2020-08-03

**Authors:** Damian Szczesny, Katarzyna Mołoniewicz, Michał J. Markuszewski, Paweł Wiczling

**Affiliations:** Department of Biopharmacy and Pharmacodynamics, Medical University of Gdańsk, 80-416 Gdańsk, Poland

**Keywords:** Erythropoietin, Osmotic pumps, Target-mediated drug disposition, Minimal effective concentration, Subcutaneous infusion

## Abstract

**Background:**

Human recombinant erythropoietin (rHuEPO) is often used in the treatment of diseases associated with a decreased production of red blood cells (RBC), such as chronic renal failure. rHuEPO is typically administered as an intravenous or subcutaneous (SC) injection every few days. The low minimum effective concentration (MEC) of rHuEPO, compared to the concentrations observed after standard doses, suggests that a low dose of the drug administered as a long-term infusion should be efficacious. This study aimed to compare the efficacy observed after a single subcutaneous administration of rHuEPO with that observed after a long-term infusion of rHuEPO via implanted osmotic pumps at a similar or lower dose.

**Materials and methods:**

In this study three rats received rHuEPO as a single SC injection at a dose of 1350 IU/kg, nine via osmotic pumps at a rate of 0.25, 0.5 and 1 IU/kg and at a total dose of 333 IU/kg, 667 IU/kg, 1333 IU/kg. Three rats served as a control group. The erythropoietin concentrations, RBC count and hemoglobin were measured.

**Results:**

An increase in RBC count and hemoglobin was observed after SC infusion of rHuEPO. The baseline corrected area under the effect curve for hemoglobin and RBC count was more than 10-times higher for the SC infusion than for a single SC administration with a comparable dose.

**Conclusions:**

This study demonstrates that administration of rHuEPO as a long-term infusion at a rate ensuring MEC allows to achieve a high efficacy of therapy using relatively small doses of the drug.

## Introduction

Erythropoietin (EPO) is a glycoprotein involved in maintaining sufficient red blood cells (RBC) production [1]. In adult humans, EPO is produced mainly by the renal cortex, which contributes to ~ 90% of the blood level of this hormone. Other organs producing EPO include liver, spleen, lungs, testis, brain, and erythroid progenitor cells. In a healthy human, the blood concentration of EPO is 10 pM (< 5–25 IU/l) [2]. It is similar to the baseline value reported for rats (5.4 IU/l) [3]. Erythropoietin is a main regulator of erythropoiesis, a process in which new erythrocytes origins from pluripotent stem cells in the bone marrow. In humans and other mammals, erythropoiesis is a slow process in which old RBCs are replaced with young reticulocytes. In some pathologic conditions, like hemorrhage or severe hemolysis, the rate of reticulocyte production may increase even eight times and an increase in EPO concentration might be 1000-fold [2, 4].

Erythropoietin binds to the specific erythroid progenitor cell surface receptor (EPO-R) to regulate bone marrow erythroid cell proliferation, differentiation, and survival. Binding to EPO-R expressed on the erythroid progenitor cells in the bone marrow has been reported as an important pathway of EPO elimination [5, 6].

Recombinant human erythropoietin (rHuEPO) is a biotechnologically derived drug widely used in the treatment of different types of anemia, like anemia associated with chronic kidney disease, in HIV-infected or cancer patients. rHuEPO is often administered as a SC or IV injection, up to three times a week [7]. It was demonstrated, that higher pharmacological response might be achieved after SC administration and divided doses [8]. This observation led to the assumption, that pharmacological response depends on the time when EPO concentration exceeds a certain level instead of peak concentration. This concentration was called “critical” by Besarab et al. [8], later renamed into “minimum effective concentration”. According to this hypothesis, prolonged administration of EPO keeps its concentration above minimum effective concentration (MEC) and can produce an equal or higher pharmacological effect, even when the total dose of EPO is lower than after bolus injection [9]. The MEC value in humans ranges from 60 to 20 mIU/ml and is close to the blood concentration of EPO [9]. The relatively low value of MEC for rHuEPO has an interesting implication for rHuEPO dosing, especially if given as a constant infusion. As an example, to achieve rHuEPO concentration in rats four-fold higher than the baseline EPO concentration (Ct = 4 × 5.4 IU/l), rHuEPO needs to be administered at a fairly small rate of 25.5 × 21.6/1000 = 0.551 IU/h/kg. In this calculation an apparent rHuEPO clearance of CL/*F* = 25.50 ml/h/kg was assumed [10]. Consequently, a rHuEPO dose of 1350 IU/kg allows one to perform an infusion for about 102 days (1350/0.551). Since under the MEC hypothesis, EPO is effective for concentrations exceeding baseline concentrations, this calculation shows that large efficacy can be achieved by administering a single rHuEPO dose over a long time.

Based on the above-mentioned premises, this study was developed to compare the pharmacological effect of EPO observed after a single SC injection at a 1350 IU/kg dose and a long-term SC infusion at a similar or lower dose. For that purpose, pharmacokinetic and pharmacodynamic (PK/PD) of rHuEPO during this infusion via implanted osmotic pumps was determined.

## Materials and methods

### Animals

Animal care and handling were carried out according to the protocol approved by the Local Ethical Committee for Animal Research at the Medical University of Gdańsk. The male Wistar rats were obtained from Tri-city’s Academic Animal Experiment Centre, Gdańsk, Poland. All animals were accommodated in the laboratory for at least 2 weeks before the start of the study. They entered the study when they were 6 weeks old and weighted ~ 300 g. Animals were fed with standard chow (Labofeed H Standard, Diet Manufacturer Morawski, Poland) contains 150 mg of iron per kilogram and water ad libitum and were housed in a room with 12/12-h light/dark cycle.

### Experimental procedures

In total 15 rats were used in this experiment. Animals were divided into five groups (*n* = 3). Erythropoietin (Epogen, 2000 IU/ml, Amgen, Thousand Oaks, USA) was administered by two routes: single bolus SC injection (1350 IU/kg, solution in 0.9% sodium chloride) in one group, and by continuous SC infusion via osmotic pumps (Alzet, model 2002, DURECT Corporation, Cupertino, USA), with following nominal rates of infusion: 1.0, 0.5 and 0.25 IU/h, for 14 days. Pumps were filled with rHuEPO solution in 0.9% sodium chloride at a total dose of 1333 IU/kg, 667 IU/kg, and 333 IU/kg. One group received a 0.9% solution of sodium chloride only and served as a control. The rates were determined based on the simulation of a pharmacokinetic model published by Woo et al. [10] to ensure the concentrations of rHuEPO 2-, 4-, and 8-times higher from the endogenous EPO concentrations. The latter was assumed to equal 5.4 mIU/ml in rats [3].

### Osmotic pumps implantation

Osmotic pumps were filled with a solution of rHuEPO after proper dilution in 0.9% sodium chloride. 2000 IU/ml primary solution of erythropoietin was diluted with 0.9% sodium chloride at a ratio of 1:1, 1:2, and 1:4. Each dilution was used for filling three osmotic pumps at a volume of 200 µl. Pumps were then implanted subcutaneously on the middle-back of an animal in sterile conditions under the anesthesia. As an anesthetic agent, 1–3% isoflurane (Forane, Abott, Kent, UK) in breathed air was used. Wounds were closed with wound clips and clips were removed after 1 week. There were no signs of infection or inflammation in the site of pump implantation.

### Pharmacokinetic and pharmacodynamic study

To determine pharmacokinetic and pharmacodynamic properties of rHuEPO, 100 μl of blood was taken before osmotic pump implantation or SC administration of rHuEPO and then after 5, 24, 48 and 72 h (SC injection group only) and 5, 10, 15, 17, 19, 22, 29 days (each group). For red blood cells and hemoglobin quantification, 50 µl of blood was taken at baseline and 2, 5, 6, 8, 10, 13, 15, 17, 20, 22, 24, 27, 29, 31, 34, 41, 48, 55, and 62 day (each group) after the beginning of the experiment. Coagulation of fresh whole blood was prevented using 1% EDTA in amounts not exceeding 10% of the blood sample by volume.

The blood was centrifuged (3500 rpm, 10 min, room temperature), serum was separated and frozen in −80 °C until erythropoietin level was determined by Quantikine IVD ELISA (Human EPO Immunoassay, R&D System Inc., Minneapolis, USA) following manufacturer instructions. Concentrations were determined based on the standard curve, linear from 2.5 to 200 mIU/ml. The detection limit was 2.5 mIU/ml. Samples with a concentration higher than 200 mIU/ml were diluted with the solvent provided by the manufacturer of assay kit. The assay did not cross-react with endogenous EPO. The conversion from IU/l to pM was made assuming 7.7 mg of rHuEPO is equivalent to 1000 IU and the molecular weight of rHuEPO of 30.4 kD [11].

Hematological analysis was done with use of ABC VET hematological analyzer (Horiba Medical Diagnostics Instruments and Systems, Montpellier, France) immediately after blood sampling.

### Statistical analysis

The calculation of AUC for noncompartmental analysis has been done by the trapezoidal method with extrapolation to infinity based on the last two observed points. The comparison of the pharmacological effect for various methods of administration and different doses of rHuEPO was based on maximal observed RBC and Hb values, and the area under the effect curve (AUCE). The latter allows the comparison of net effect of drug [12]. AUCE was calculated by the trapezoidal integration over the time of measurements.

The difference between the control and rHuEPO treated rats was tested using Kruskal–Wallis test (a nonparametric version of one-way ANOVA) with Dunn-Bonferroni post hoc method for multiple comparisons. *p *value < 0.05 was accepted as significant. Statistical analysis was performed in R environment (R Core Team 2019) using dunn.test package version 1.3.5.

## Results

In this study rats received rHuEPO as a single subcutaneous injection and as a subcutaneous infusion at three different rates of 0.25 IU/h, 0.5 IU/h, 1 IU/h. Implantation of the osmotic mini-pumps under the skin fold located on the neck of the animal was a relatively simple procedure without any complications. The wound healed quickly and the pump itself did not interfere with the normal functioning of the animal.

**Fig. 1:**
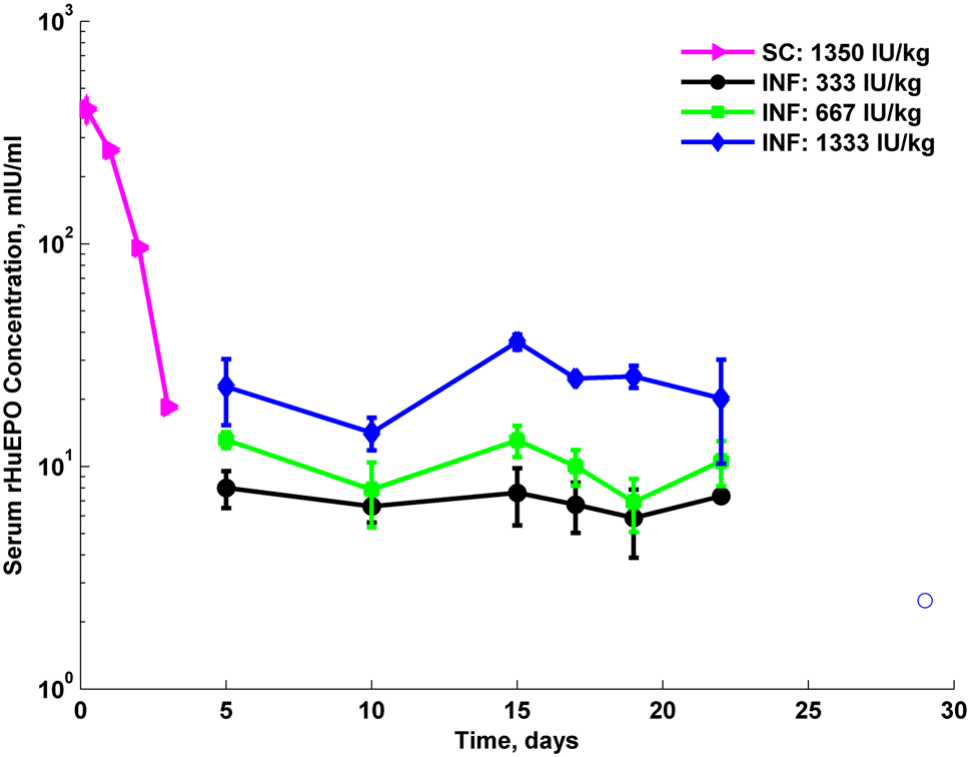
Time course of rHuEPO serum concentrations after single SC injection of 1350 IU/kg, and three subcutaneous infusions at nominal rates 0.25, 0.5 and 1 IU/h. The mean observed data are represented as solid symbols with SD error bars. The open symbol denotes concentration below lower limit of quantification of the assay

The rHuEPO serum concentrations are shown in Fig. 1. When using the osmotic pump in vivo concentrations increased and sustained steady state values from day 5 to day 22. The basic PK parameters were calculated by noncompartmental analysis and are summarized in the Table 1. For SC infusion groups, steady-state concentrations were 7.03, 10.3 and 23.1 mIU/ml after 333, 667 and 1333 IU/kg dose of rHuEPO. These values are much lower than concentrations observed within first two days after single SC injection of rHuEPO. Apparent clearances, calculated as a ratio of a nominal rate by steady-state concentration (for infusion groups) or dose over AUC (SC injection) were 36.3, 49.0, 43.7 ml/h for infusion with rate of 0.25, 0.5 and 1.0 IU/h, respectively, and 29.7 ml/h for rats injected with rHuEPO.

**Table 1:** The basic PK parameters and their standard deviations obtained after SC infusion and SC injection of rHuEPO in rats

Group, IU/kg	*C*_ss_, mIU/ml	*R*_0_, IU/h	CL/*F*, ml/h	CL/*F*/BW, ml/h/kg
INF: 333	7.03 (1.3)	0.25	36.3 (6.7)	121.2 (22.3)
INF: 667	10.3 (1.1)	0.5	49.0 (5.4)	163.5 (17.8)
INF: 1333	23.1 (2.7)	1	43.7 (5.0)	145.6 (16.8)
SC: 1350	–	–	29.7 (3.7)	99.1 (12.3)

The apparent clearance were calculated as a ratio of nominal rate (*R*_0_) and steady-state concentrations (*C*_ss_), for single injection as a dose over AUC. Data are presented as mean (SD)

**Fig. 2:**
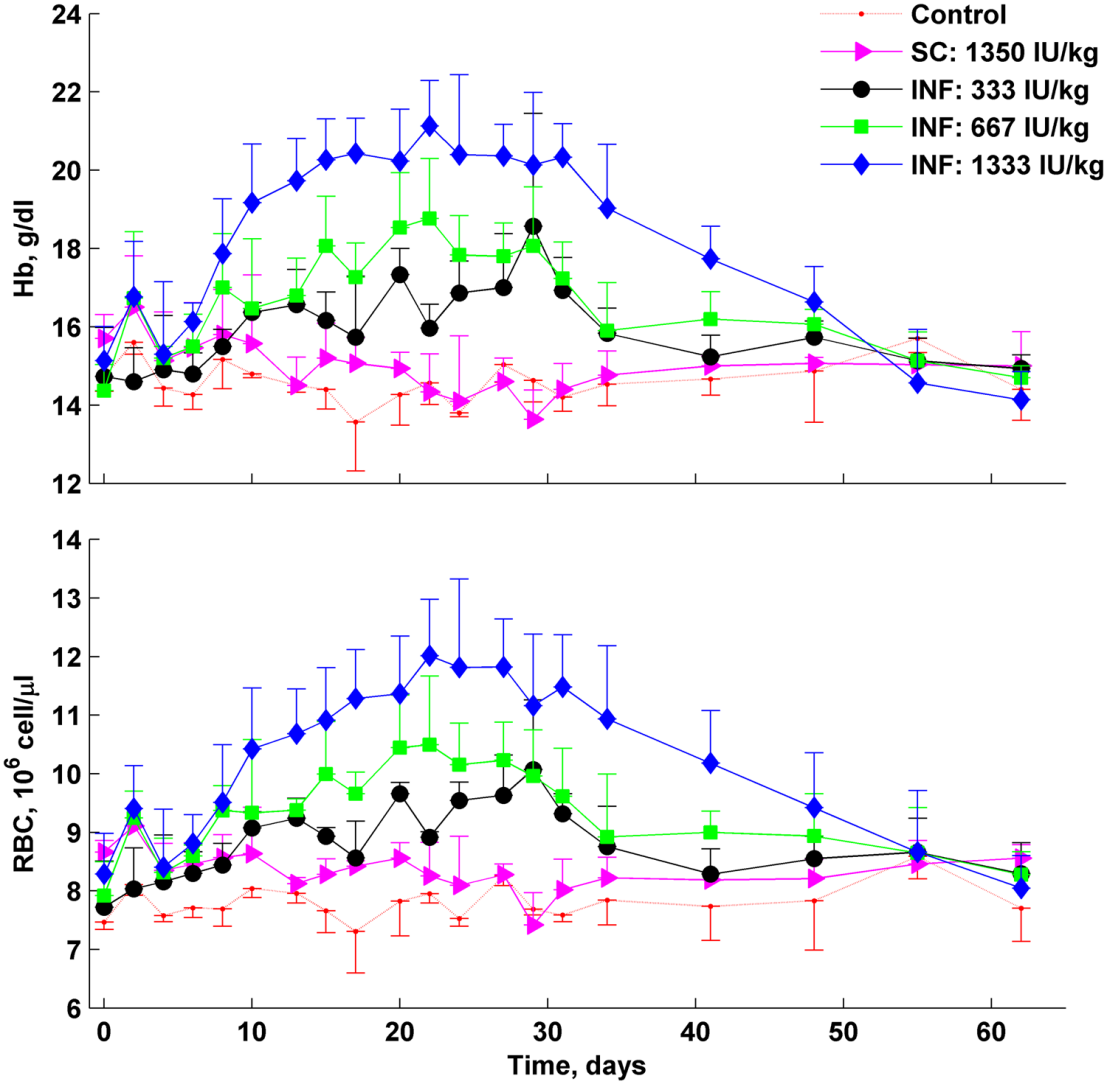
Time course profiles of mean hemoglobin concentrations and RBC count for control, single SC injection of 1350 IU/kg, and three subcutaneous infusions at nominal rates of 0.25, 0.5 and 1 IU/h. The mean observed data are represented as solid points with SD error bars

To investigate pharmacodynamics of rHuEPO after SC infusion and injection, red blood cells count (RBC) and hemoglobin concentration (Hb) were measured. Changes in RBC and Hb in rats are shown in Fig. 2. The results are summarized in Table 2.

**Table 2:** The area under the effect curve (AUCE) and maximal value for Hb and RBC in control and rHuEPO treated rats

Group	AUEC for RBC, 10^6^ cells/µl·day	AUEC for Hb, g/dl·day	Max RBC, 10^6^ cells/µl	Max Hb, g/dl
Overall test (*n* = 15)	Chi-sq = 12.43, *df* = 4 *(*p* = 0.01)	Chi-sq = 12.1, *df* = 4 *(*p* = 0.02)	Chi-sq = 10.9, *df* = 4 *(*p* = 0.03)	Chi-sq = 10.4, *df* = 4 *(*p* = 0.03)
Control (*n* = 3)	488 (± 20) 497 (473–500)	911 (± 18) 920.6 (898–921)	8.6 (± 0.4) 8.7 (8.3–8.8)	16.0 (± 0.4) 15.9 (15.9–16)
INF: 333 IU/kg (*n* = 3)	543 (± 17) 539 (532–556) (*p* = 0.34)	981 (± 16) 984 (960–993) (*p* = 0.60)	10.2 (± 1.0) 9.8 (9.5–11.0) (*p* = 0.50)	18.7 (± 2.8) 17.2 (17.0–20.7) (*p* = 0.41)
INF: 667 IU/kg (*n* = 3)	571.6 (± 37) 569 (544–600) (*p* = 0.07)	1021 (± 40) 1020 ( 991–1051) (*p* = 0.18)	10.6 (± 1.0) 10.3 (9.9–11.4) (*p* = 0.11)	18.9 (± 1.5) 18.6 (18.0–20.0) (*p* = 0.14)
INF: 1333 IU/kg (*n* = 3)	627 (± 56) 650 (584–663) *(*p* = 0.0096)	1109 (± 67) 1135 (1059–1152) *(*p* = 0.017)	12.2 (± 1.0) 12.8 (11.5–12.8) *(*p* = 0.0096)	21.3 (± 1.2) 21.9 (20.4–22.0) *(*p* = 0.011)
SC: 1350 IU/kg (*n* = 3)	516 (± 5) 515 (512–520) (*p* = 1.0)	928 (± 24) 921 (911–946) (*p* = 1.0)	9.56 (± 0.3) 9.54 (9.34–9.77) (*p* = 1.0)	17.2 (± 0.7) 17.2 (16.8–17.6) (*p* = 1.0)

Data are presented as mean (± SD) and medians (quartiles, 25th–75th percentile). The Kruskal–Wallis rank sum test with Dunn–Bonferroni post hoc method was used to compare groups. Asterisk denotes statistically significant increase over control values

We observed a dose-dependent increase of RBC count and hemoglobin level upon mini-pump implantation. Rats administered with rHuEPO at a rate of 0.25 IU/h showed an increase in RBC and Hb to a maximum of 10.2 × 10^6^ cells/µl and 18.7 g/dl, respectively. The 0.5 IU/h group reached maximal RBC and Hb responses of 10.6 × 10^6^ cells/µl and 18.9 g/dl, and the 1 IU/h group reached maximal RBC and Hb responses of 12.2 × 10^6^ cells/µl and 21.3 g/dl. The RBC and Hb increased to about 30 days after pump implantation. Between day 30 and day 60 a linear decline of RBC and Hb toward the baseline was observed due to the discontinuation of rHuEPO administration.

The maximum value for RBC and Hb responses after a single SC injection was recorded 48 h after administration of rHuEPO and equaled to 9.56 × 10^6^ cells/µl and 17.2 g/dl. It is much lower than the values observed for rats administered via subcutaneous infusion.

Table 2 presents the AUCE values for RBC count and hemoglobin. AUCE for RBC increased by 28 × 10^6^ cells/µl·day for SC injection and by 56, 84, 139 × 10^6^ cells/µl·day for administrations with rates of 0.25, 0.5 and 1 IU/h. AUCE for hemoglobin showed a comparable pattern. It increased by 17 g/dl·day for subcutaneous injection, and by 71, 110 and 198 g/dl·day for administrations with rates of 0.25, 0.5 and 1 IU/h.

## Discussion

The rHuEPO PK in rats is usually described by a two compartmental model with parallel first-order elimination and nonlinear Michaelis–Menten elimination. Absorption after SC injection is a process that consists of an initial, zero-order, and a subsequent, first-order phase [10, 13]. Also, a target-mediated drug disposition approach was used to describe the pharmacokinetics of rHuEPO. In this case, binding of EPO to the receptor (EPO-R) and internalization of this complex was necessary to capture the nonlinear pharmacokinetics of rHuEPO observed for low doses [14]. For drugs exhibiting this type of disposition, an increase in apparent clearance with an increase in dose is observed [15, 16]. In our study, apparent (due to unknown bioavailability) clearance was few-fold higher than observed in the literature. It could be explained by the difference in study design or different bioavailability after the long-term SC injection of the drug.

Pharmacokinetics of EPO after SC administration is usually described by flip-flop kinetics, implying the rate of elimination is faster than the rate of absorption. This phenomenon leads to a slow decline of the drug in plasma [10]. This phenomenon might explain the fact, that even at 22 days after implantation of the osmotic pumps rHuEPO was still present in rats’ blood.

In a pharmacodynamic part of our work, the effect of continuous infusion of rHuEPO on RBC count and hemoglobin concentration was tested. We showed that administration of rHuEPO as a subcutaneous infusion is efficacious. In a study by Ait-Oudhia et al. [17], the peak number of RBC was 11.05·10^6^ cells/µl and the highest observed Hb concentration was 24.27 g/dl. It was observed after repeated three-times-per-week rHuEPO administration at a dose of 1350 IU/kg for 6 weeks. In our study, we found the highest responses (12.2 × 10^6^ cells/µl and 21.3 g/dl, respectively) after infusion with a rate of 1 IU/h (total dose of 1333 IU/kg), but even after the slowest infusion (0.25 IU/h, 333 IU/kg), peak RBC count and Hb were 10.2 × 10^6^ cells/µl and 18.7 g/dl. This demonstrates, that a similar pharmacodynamic effect might be obtained with a lower amount of rHuEPO when it is delivered as a continuous infusion instead of repeated injection.

In our study design, we performed every procedure on both, treated and control groups, so a possible increase in hemoglobin and red blood cells due to the sampling should be detected in the control group. Rats in the control group had approximately constant hemoglobin content (14.6 ± 0.14 g/dl) during the whole experiment which is expected for 12-week old rats [10]. This indicates a lack of sampling effects on PD responses.

It has been observed that smaller doses of rHuEPO are required to maintain the same levels of hematocrit after subcutaneous injections than after intravenous injections [8, 9]. Also for intravenous administration, about ten times higher peak concentrations are typically observed than after subcutaneous injection. This suggests that the hematological effect of rHuEPO is not driven by peak concentrations, but depends more on the duration of the drug concentration above the "critical concentration" or minimum effective concentration (MEC) [8]. The results presented in this paper confirm the thesis that hematological effect is largely dependent on how long the EPO remains in the circulation. Similar results to observed in this study might also be expected for pegylated forms of EPO and modern medicines, especially from the group of CERA (continuous erythropoietin receptor activator), due to the similar mechanism of action [20].

The stimulatory effects of erythropoietin are not solely dependent on the plasma concentration, but also dependent on the mechanism of EPO binding to its receptor. To date, it is unclear how long the ligand molecule is bound with the EPO-R. It is known that the amount of the erythropoietin receptor is relatively low on progenitor cells (about 200 receptors per cell), that these receptors have a different affinity for EPO, and that only about 20–30% of receptor occupancy is required to stimulate erythropoiesis [18, 19].

rHuEPO is used in the treatment of anemia, especially if it is associated with renal failure, AIDS, or during cancer chemotherapy [21, 22]. In the course of cancer, anemia affects about 90% of patients, of which 60% require a blood transfusion. Treatment with rHuEPO is an alternative to transfusion in cancer patients. Patients are usually receiving rHuEPO injection 3 or 4 times a week. In recent years, however, are growing doubts about the appropriateness of the use of erythropoietin in cancer patients in the view of reported decreased survival of cancer patients treated with EPO [22, 23]. It has been shown that in some cases administration of exogenous erythropoietin can stimulate the growth of tumors. This is probably related to the high doses of EPO and the presence of EPO-R receptors on the surface of certain cancer cells [24, 25]. After binding the ligand, EPO-R can stimulate cell growth. EPO-R is also present on the surface of endothelial cells, which may suggest that there is a risk of stimulation of angiogenesis and tumor progression [22]. Based on the study it can be hypothesized that frequent administration of low doses of rHuEPO can be considered in the treatment of anemia in the course of neoplastic disease. It should lead to the same or higher efficacy and decreased risk of side effects (these driven by high EPO concentrations). Nevertheless, this phenomenon needs to be confirmed in appropriate clinical trials.

## Conclusion

In this work, we have shown that the administration of rHuEPO by a continuous subcutaneous infusion is highly effective in rats. The same dose administered as an infusion leads to a pharmacological effect much greater than observed after single subcutaneous administration. It is possible to administer frequent low doses of rHuEPO and retain high efficacy of therapy. The dose leading to concentrations exceeding the minimum effective concentration results in an increase in hematological parameters, such as the level of red blood cells and hemoglobin. The use of lower doses might be beneficial due to decreased risk of side effects and reduction of treatment costs if confirmed in humans.
